# Metal–Organic Frameworks, Covalent Organic Frameworks, and Metal–Covalent Organic Frameworks for Aqueous PFAS Adsorption: Synthesis, Structural Engineering, and Remediation Performance

**DOI:** 10.3390/ma19163470

**Published:** 2026-08-17

**Authors:** Liu-Feng Yu, Min-Min Tang, Jie Zou, Wei Gao, Yi Zhang, Hong-Zhen Lian

**Affiliations:** 1 Key Laboratory of Food Contact Materials Safety, State Administration for Market Regulation, Jiangsu Product Quality Testing & Inspection Institute, Nanjing 210007, China; 6240112182@stu.jiangnan.edu.cn (L.-F.Y.); tangminmin.1988@163.com (M.-M.T.); 13951700473@139.com (J.Z.); jszjgaowei@126.com (W.G.); 2 State Key Laboratory of Food Science and Resources, School of Food Science and Technology, Jiangnan University, Wuxi 214122, China; 3 State Key Laboratory of Analytical Chemistry for Life Science, School of Chemistry & Chemical Engineering and Centre of Materials Analysis, Nanjing University, Nanjing 210023, China

**Keywords:** per- and polyfluoroalkyl substances, metal–organic frameworks, covalent organic frameworks, metal–covalent organic frameworks, adsorptive removal, water remediation

## Abstract

Per- and polyfluoroalkyl substances (PFAS) represent a critical class of persistent environmental pollutants that challenge conventional remediation technologies. Metal–organic frameworks (MOFs), covalent organic frameworks (COFs), and the emerging metal–covalent organic frameworks (MCOFs) have attracted growing interest as tunable porous platforms for removing per- and polyfluoroalkyl substances (PFAS) from water. This review critically summarizes recent advances in the synthesis, structural characterization, and adsorptive performance of these three framework families. We examine key factors affecting adsorption, including solution chemistry and PFAS chain length, and elucidate the underlying mechanisms—hydrophobic/fluorophilic interactions, electrostatics, anion exchange, and coordination—through combined experimental and computational evidence. A comparative assessment evaluates adsorption capacities, kinetics, stability, and regenerability, identifying the distinctive advantages of each material class. Practical applications in fixed-bed columns and magnetic composites are also discussed. Finally, we highlight persistent challenges, particularly in the removal of ultrashort-chain PFAS, and outline future directions involving machine learning, defect engineering, and dual-functional adsorptive–catalytic platforms to guide rational adsorbent design.

## 1. Introduction

Per- and polyfluoroalkyl substances (PFAS) are a diverse group of synthetic fluorinated organic compounds containing fully or partially fluorinated alkyl chains [1]. Since their commercial introduction in the mid-twentieth century, PFAS have been widely used in aqueous film-forming foams, fluoropolymer production, metal plating, textiles, food-contact materials, and surface-treatment applications because of their thermal and chemical stability and their water- and oil-repellent properties [2]. Based on their degree of fluorination and functional groups, PFAS can be categorized into perfluoroalkyl acids (PFAAs), perfluoroalkyl sulfonamides, fluorotelomers, and other subclasses (Table 1). Owing to the exceptionally high bond energy of the C–F bond, PFAS exhibit remarkable hydrophobicity, oleophobicity, thermal stability, and chemical inertness, rendering them resistant to hydrolysis, photolysis, or biological degradation under natural conditions [2,3]. This extreme stability endows PFAS with the typical characteristics of persistent organic pollutants [4]. PFAS enter aquatic environments through releases during manufacturing, product use and disposal, firefighting activities, wastewater discharge, and landfill leachate.

The global scale of PFAS contamination is staggering. A meta-analysis of groundwater data from 20 countries reported perfluorooctanoic acid (PFOA) concentrations ranging from <0.03 ng/L to ~7 mg/L, and perfluorooctane sulfonate (PFOS) concentrations from 0.01 ng/L to ~5 mg/L [5]. The highest PFAS concentration in groundwater, ~15 mg/L, was reported for the replacement compound 6:2 fluorotelomer sulfonate (6:2 FTS). A clear disparity exists in monitoring coverage, with studies concentrated in developed regions, whereas systematic assessments are critically lacking in many developing areas [6]. Groundwater—which supplies a substantial portion of drinking water globally—has been identified as a major pathway for human exposure [5,7]. The ubiquitous distribution of PFAS, including in areas far removed from specific sources, gives rise to a “background” concentration that must be accounted for in risk assessments [5]. Information from 461 reported PFAS-contaminated groundwater sites indicates that firefighting training activities are a major source, accounting for 41% of cases, followed by other fluorinated industrial activities, landfill leachate, and wastewater leakage [6].

The public health concerns driving regulatory actions are substantial and well-documented. Epidemiological studies have revealed associations between exposure to specific PFAS and a variety of health effects, including altered immune and thyroid function, liver disease, lipid and insulin dysregulation, kidney disease, adverse reproductive and developmental outcomes, and cancer [8,9]. The most significant health effects associated with exposure to long-chain PFASs (such as PFOA and PFOS) include lipid disorders, hypertension, diabetes mellitus, thyroid disorders, infertility, cancer, obesity, neurodevelopmental issues, cardiovascular diseases, and kidney and liver disorders [10,11]. Legacy PFAS such as PFOA and PFOS have been linked to an increased risk of kidney and testicular cancer [8]. Nearly all of the global population has some detectable PFAS in their bodies [9].

In response to mounting evidence of widespread contamination and health risks, regulatory frameworks have evolved rapidly [12,13]. The U.S. Environmental Protection Agency (EPA) issued its first-ever national primary drinking water regulation for PFAS on 10 April 2024, establishing legally enforceable individual maximum contaminant levels (MCLs) for five PFAS chemicals: PFOA, PFOS, PFHxS, PFNA, and HFPO-DA (GenX) [14]. PFOA and PFOS were set at four parts per trillion (ppt) each, with water systems required to comply by 2029 [14]. The European Union’s recast Drinking Water Directive (2020/2184), effective from January 2026, mandates harmonized monitoring of PFAS levels and establishes a two-tier limit: 0.1 μg/L (100 ng/L) for the sum of 20 PFAS compounds and 0.5 μg/L (500 ng/L) for total PFAS [15]. At the international level, PFOS and PFOA have been listed under the Stockholm Convention on Persistent Organic Pollutants, subjecting them to global production and use bans with limited exemptions [16]. The World Health Organization (WHO) has been developing background documents for its Guidelines for Drinking-water Quality focusing on PFOS and PFOA [17].

The convergence of these regulatory pressures—enforceable MCLs, harmonized monitoring requirements, and international treaty obligations—has created an urgent demand for effective PFAS removal technologies [18]. Conventional adsorbents such as granular activated carbon and ion-exchange resins exhibit inherent limitations, including slow adsorption kinetics, poor removal efficiency for short-chain PFAS congeners, and unsatisfactory regeneration capabilities [19]. These technological gaps have catalyzed intensive research into advanced porous materials—particularly metal–organic frameworks (MOFs), covalent organic frameworks (COFs), and the emerging metal–covalent organic frameworks (MCOFs)—as promising adsorbent platforms capable of achieving the selectivity, capacity, and stability required for next-generation PFAS remediation.

## 2. Design, Synthesis, and Structural Chemistry of Framework Adsorbents

### 2.1. MOFs

MOFs represent a class of crystalline porous materials constructed from inorganic metal ions or metal-oxo clusters interconnected by polydentate organic linkers via coordination bonds [20]. The architectural diversity of MOFs arises from the virtually unlimited combinations of metal nodes and organic struts, enabling the rational design of frameworks with predetermined topologies and pore geometries [21]. The metal nodes commonly employed for PFAS adsorption applications include zirconium (Zr^4+^), iron (Fe^3+^), aluminum (Al^3+^), and copper (Cu^2+^), among which Zr-based MOFs—particularly the UiO-66 series—have received the most extensive attention due to their exceptional chemical and thermal stability [22,23]. The UiO-66 topology features a face-centered cubic arrangement of Zr_6_O_4_(OH)_4_ clusters coordinated to 1,4-benzenedicarboxylate (BDC) linkers, resulting in a microporous structure with tetrahedral and octahedral cages of approximately ~11 Å and ~21 Å in diameter, respectively [24]. The MIL (Materials of Institute Lavoisier) family, particularly MIL-101(Cr), offers large mesoporous cages (~29–34 Å) and a reported model-derived apparent surface area of approximately 5900 ± 300 m^2^/g, calculated from N_2_ adsorption data using the Langmuir model, making this framework attractive for accommodating bulky PFAS molecules [25,26]. Zeolitic imidazolate frameworks (ZIFs), exemplified by ZIF-8 with a sodalite-type topology constructed from Zn^2+^ ions and 2-methylimidazolate linkers, feature narrow apertures (~3.4 Å) that impose size-exclusion effects [27]. This property is speculatively expected to hinder the uptake of long-chain PFAS, while it may tentatively permit access for short-chain congeners. The structural diversity of these representative MOFs is summarized in Table 2.

The synthesis of MOFs has evolved from conventional solvothermal methods to a broader spectrum of preparative routes, each offering distinct advantages in controlling crystallinity, particle size, and morphological features [28]. Solvothermal synthesis, typically conducted in polar organic solvents (e.g., N,N-dimethylformamide, dimethyl sulfoxide, or methanol) at elevated temperatures (80–200 °C) and autogenous pressures, remains the most widely employed approach due to its versatility and the high crystallinity of the resulting products [20]. Temperature, reaction time, solvent composition, and the concentration of modulators (e.g., formic acid, acetic acid, or trifluoroacetic acid) critically influence the crystallite size, defect density, and textural properties of MOFs [29]. Electrochemical synthesis offers an alternative pathway wherein metal ions are continuously supplied from sacrificial anodes under an applied electric potential, enabling rapid and scalable production while minimizing the use of metal salts and reducing reaction times from hours to minutes [30]. Mechanochemical synthesis, performed via ball-milling of solid precursors without or with minimal solvent, has gained traction as a sustainable approach that yields nanoscale MOF crystallites with controlled particle size distribution, though careful optimization is required to maintain long-range crystalline order [31]. The selection of synthetic methodology directly impacts the physicochemical properties of MOF adsorbents, including surface area, pore accessibility, and the density of active sites, which collectively determine their PFAS adsorption performance [32].

**Table 2:** Representative MOFs for PFAS adsorption: performance summary.

MOFs	Target PFAS	Surface Area (m^2^/g)	pH of System	Equilibrium Time (min)	Capacity (mg/g)	Best Removal Rate (%)	Regeneration	Ref.
MIL-101(Cr), MIL-101(Cr)-NH_2_, MIL-101(Cr)@AC	PFOS	2420; 433; 6955	4.0; 2.0; 4.0	15; 30; 120	10.1; 2.8; 25.7	71.2; 20.5; 80.4	~40% after 4 cycles; Not tested (low performance); ~50% after 4 cycles	[33]
MOF-808	PFOS	1610	4.1–5.4	30	939	~100	-	[34]
NU-1000	PFSAs (C4–C8), PFCAs (C1–C9)	2255	-	10	400–620 (PFSA); 201–604 (PFCAs)	75–98	>86% recovery after 5 cycles	[35]
SCU-8	PFOS	1360	-	2	147.6	96	~44% recovery after 4 cycles	[36]
UiO-66 (defective)	PFOS, PFBS	1125	5.0	120	~620; ~408	~90	-	[37]
ZIF-8@Cu	PFOS	-	~7.0	24 h	0.08	98	-	[38]
Basolite A100	PFOA	-	3.5	240	169.2	~70	>60% recovery after 4 cycles	[39]
MOF-808@Chitin Cryogel	PFOA, PFDA, PFOS	~23	6.8	~30	496 (PFOA); 690 (PFDA); 355 (PFOS)	~95 (PFOA); ~86 (PFDA); ~78 (PFOS)	~85–90% after 5 cycles	[40]
MIL-101(Cr)-DMEN/-QDMEN	PFOA	1692/1530	5.0	~60	~535 (DMEN); ~754 (QDMEN)	-	~90% retained after 3 cycles	[41]
PCN-222(Fe)	PFOS	1948	~5.0	30	2257	-	-	[42]
UiO-67-F_2_	PFOA	1966	-	~150	Up to 3060	-	-	[43]
Zr-PTA1	PFOA, PFOS	2045	-	<3	2945 (PFOA); 2322 (PFOS)	>99 (PFOA); >99 (PFOS)	~100% after 10 cycles	[44]
Zr-PTA2	PFBA, PFBS	2106	-	~10	375 (PFBA); 414 (PFBS)	>99 (PFBA); >99 (PFBS)	~100% after 10 cycles	[44]
MOF 5	PFBS, PFOS, PFOA, PFBA	394	-	~30	565–609	>95 (PFBS); >95 (PFOS); ~100 (PFOA); ~85 (PFBA)	Excellent (>15 cycles)	[45]
Ce-M@D-sponge	PFOA, PFOS, PFHxA, PFHxS, PFBA, PFBS	N/A (composite)	4	~120	Up to 10	~99 (PFOA, PFOS); ~94–96 (PFHxA, PFHxS, PFBA, PFBS)	>90% after 8 cycles	[46]

### 2.2. COFs

COFs are a class of crystalline porous polymers constructed from organic building blocks connected through robust covalent bonds, offering a periodic network architecture entirely devoid of metal components [47]. Unlike MOFs, whose structural integrity relies on reversible coordination bonds, COFs are stabilized by strong covalent linkages, which confer enhanced chemical stability in aqueous environments, broader pH tolerance, and superior thermal resistance [48]. The structural design of COFs proceeds through reticular chemistry principles, wherein predesigned organic monomers undergo condensation reactions to form extended two-dimensional (2D) or three-dimensional (3D) frameworks [49]. The most extensively investigated COF subclasses for PFAS adsorption include imine-linked COFs (formed via Schiff-base condensation of aldehydes and amines), β-ketoenamine-linked COFs (formed via reversible enol-keto tautomerization followed by irreversible enol-to-enamine conversion), and triazine-based COFs (constructed via trimerization of nitrile-containing monomers) [50] (Table 3). Among these, β-ketoenamine-linked COFs such as TpPa (derived from 1,3,5-triformylphloroglucinol and p-phenylenediamine) and TpBD (derived from 1,3,5-triformylphloroglucinol and benzidine) have demonstrated exceptional hydrolytic stability, rendering them particularly suitable for aqueous-phase PFAS remediation [51].

A defining structural characteristic of 2D COFs is the layered stacking of extended π-conjugated sheets via non-covalent interactions, resulting in well-defined 1D channels with uniform pore sizes typically ranging from 1.5 to 3.5 nm [47,52]. The interlayer stacking orientation—eclipsed (AA) versus staggered (AB)—critically governs the effective pore aperture and the accessibility of functional groups lining the channel walls [49]. The pore architecture can be systematically tuned by varying the geometry and size of the building blocks, enabling precise molecular sieving effects [53]. Furthermore, the organic nature of COFs permits the straightforward introduction of functional moieties (e.g., –F, –CF_3_, –NH_2_, –SO_3_H) either through the selection of pre-functionalized monomers or via post-synthetic modifications, thereby enabling tailored host–guest interactions with PFAS molecules [50,54].

The synthesis of high-quality COFs necessitates a delicate balance between reversibility and irreversibility in bond formation to achieve both high crystallinity and structural robustness [55]. Solvothermal synthesis, performed in sealed ampoules or autoclaves with mixed solvents (e.g., 1,4-dioxane/mesitylene, 1,4-dioxane/ethanol) at temperatures between 80 and 150 °C, remains the benchmark method for producing highly crystalline COF powders [47]. The addition of modulators such as aniline, acetic acid, or water can significantly enhance crystallinity by regulating the nucleation and growth kinetics [56]. However, the extended reaction times (typically 2–7 days) and the use of toxic solvents limit the scalability of solvothermal COF synthesis [48]. Recent advances have introduced room-temperature interfacial synthesis strategies, wherein COF films or powders are generated at liquid–liquid or liquid–solid interfaces under ambient conditions, dramatically reducing synthesis time to hours while achieving comparable surface areas (1500–2500 m^2^/g) [57]. These emerging synthetic routes, along with microwave-assisted and mechanochemical approaches, are progressively addressing the scalability challenges while maintaining the structural integrity and textural properties essential for PFAS adsorption [58,59].

**Table 3:** Representative COFs for PFAS adsorption: performance summary.

COFs	Target PFAS	Surface Area (m^2^/g)	pH of System	Equilibrium Time (min)	Capacity (mg/g)	Removal Rate (%)	Regeneration	Ref.
TAB-BD COF@ chitosan	PFOA	8.2	5.0	360	~1159	>80%	~75% after 5 cycles	[59]
TAPB-TFB	PFOA, PFOS	202	-	10	-	90 (PFOA); 99 (PFOS)	-	[60]
COF-TpBd, TpHd, TpPa, TpDt, TpQd	PFBA, PFBS, PFHxA, PFHxS, PFOA, PFOS	49.5–903.0	4.4	24 h	Up to 38 (PFOS on TpDt)	-	~76% after 6 cycles	[61]
COF-300, -methyl, -dimethyl	PFOA, PFBS, PFBA	1359 (COF-300)	6	18 h	259 (PFOA)	~100 (PFOA, PFBS); >80 (PFBA)	Not regenerable	[62]
X%[NH_2_]-COF	GenX, PFOA, PFOS, PFHxS, PFBS, PFBA	~1000	7.5	1–30	200 (GenX)	>90 (12 of 13 PFAS); 63 (PFBA)	-	[63]
COF-FxNy	PFOS, PFHxS	17.2–556.0	6	6 h	439 (PFOS); 120 (PFHxS)	~98 (PFOS); ~95 (PFHxS)	Stable after 5 cycles	[64]
FSQ-1	PFOS, PFOA, GenX, F-53B, PFOSAmS, 6:2 FTSA	1221	-	15	338–375 (for 6 PFAS)	>85 (for 24 PFAS)	Maintained high performance after multi-cycle	[65]
COF-F, COF-S, COF-FS, COF-FS-NH_2_	PFOA, HFPO-TA, C7 HFPO-TA, GenX	68.2–670.6	5	180	0.331 mmol/g (PFOA); 0.289–0.319 mmol/g (HFPO-TA)	>90 (PFOA, HFPO-TA); ~81 (GenX)	Stable after 3 cycles	[66]
CTFs	PFOA, PFOS, PFHxS, PFBS, PFHxA, PFBA, OBS, 6:2 FTS, 6:2 FTAB	Up to 3391.6	5	30	323 (PFOA)	91–100 (for 9 PFAS in DI water)	>90% after 5 cycles	[67]
TpBTFDA	PFOA, PFOS, PFNA, PFDA, PFUnDA, PFDoDA, PFHxS, 6:2 FTS	1395	3.0	60	379–521 (for 8 PFAS)	>90	>85% after 10 cycles	[68]
MELEM-COF	PFOA, PFOS, PFDA, PFNA, PFHxA, PFBS, PFBA, GenX	1272	-	30	2500 (PFOA)	>98 (for 6 PFAS at 20 ppb)	>99% after 4 cycles	[69]
Hollow Cys-COF	PFOS, PFOA, PFOPA	408	-	1	580 (PFOS); 577 (PFOA); 473 (PFOPA)	>99 (PFOS); >98 (PFOA); >95 (PFOPA)	Reusable after regeneration	[70]
QA-F-COF + magnetic SPs	PFOA (plus 17 PFAS)	480.4	-	0.5	631 (PFOA)	~100 (for 13 PFAS)	~100% after 5 cycles	[71]
FeOCl@COF	HFPO-TA, OBS	82.9	5	120	276 (HFPO-TA); 567 (OBS)	~99 (OBS)	~90% after 10 cycles	[72]
TpPa-SO_3_H COF membrane	PFOS, PFOA, PFBS, PFBA	75.8 (film)	7	-	-	>99 (PFOS); ~95 (PFOA); ~90 (PFBS, PFBA)	Stable over 4 days	[73]

### 2.3. Metal–Covalent Organic Frameworks (MCOFs)

Metal–covalent organic frameworks (MCOFs), also denoted as M-COFs or metalated COFs, represent an emerging subclass of framework materials that integrate coordinatively bound metal centers within the covalently bonded organic backbone, thereby bridging the structural and functional attributes of MOFs and COFs [74]. It is essential to establish a clear distinction: MOFs are coordination polymers wherein metal clusters serve as integral nodal components of the framework, COFs are purely organic crystalline networks stabilized by covalent bonds, and MCOFs are COF-based architectures that incorporate metal ions as peripheral substituents or chelated species within the organic struts without disrupting the covalent framework topology. This hierarchical hybridization confers upon MCOFs the dual advantages of high chemical stability (inherited from the covalent COF backbone) and accessible metal active sites (reminiscent of MOF functionality), rendering them particularly promising for PFAS capture applications where both hydrophobic interactions and specific metal–anion coordination are desirable [75].

The synthetic strategies for MCOFs can be broadly classified into two categories. The first approach, bottom-up metalation, involves the condensation of metal-containing organic monomers (i.e., pre-metalated building blocks) with complementary co-monomers to construct the framework in a single step. This strategy is particularly well-suited for porphyrin- and phthalocyanine-based COFs, wherein metal ions (e.g., Fe^3+^, Ni^2+^, Co^2+^, Zn^2+^, Cu^2+^) are pre-inserted into the macrocyclic cavities of tetrapyrrole derivatives prior to polymerization [76]. The second and more versatile approach, post-synthetic metalation (PSM), entails the introduction of metal ions into pre-formed COF skeletons through coordination to accessible chelating sites (e.g., bipyridine, phenanthroline, imine nitrogen, or porphyrin cores) after framework assembly [77]. PSM offers the advantage of preserving framework crystallinity while enabling systematic variation in metal species without re-optimizing the entire synthetic protocol. Additionally, PSM permits the partial metalation of available coordination sites, offering tunable metal loading densities.

Despite the considerable promise of MCOFs, their development for PFAS adsorption remains nascent, and several significant challenges must be addressed. Foremost among these is the inherent trade-off between high crystallinity and high metal loading density [75]. Excessive metalation often introduces structural distortions or reduces the long-range order of the framework, as coordinated metal ions may disrupt the interlayer π–π stacking interactions that underpin the crystallinity of 2D COFs [77]. This structural compromise is often accompanied by diminished surface area and pore volume, which may counteract the anticipated benefits of enhanced metal-specific PFAS binding. Future research must therefore focus on optimizing metalation conditions—including metal precursor selection, reaction temperature, metal-to-ligand stoichiometry, and coordination kinetics—to achieve frameworks that simultaneously exhibit high crystallinity, substantial metal loading, and preserved textural properties. Additionally, the stability of the metal coordination sites under aqueous conditions and in the presence of competing anions (such as sulfate, chloride, and bicarbonate) requires thorough investigation to ensure sustained performance in real water matrices.

### 2.4. Green and Scalable Synthesis Considerations

The translation of MOF, COF, and MCOF adsorbents from laboratory-scale research to practical water treatment applications is critically contingent upon the development of environmentally benign and economically viable synthetic methodologies. The materials journal audience, including reviewers and editors of Materials, places substantial emphasis on the practical feasibility and sustainability of synthesis routes, as the environmental footprint and production cost of the adsorbent directly influence its technological relevance [78,79]. Conventional solvothermal syntheses, while effective in producing high-quality crystalline frameworks, often rely on toxic organic solvents (e.g., DMF, DMAc, 1,4-dioxane), high temperatures, and extended reaction times, which collectively render them unsuitable for large-scale production from both economic and environmental perspectives.

In response to these limitations, significant research efforts have been directed toward developing green synthesis strategies. Aqueous-phase synthesis represents a particularly attractive approach, wherein water serves as the sole or primary solvent for framework assembly. For MOFs, water-based synthesis has been demonstrated for several zirconium- and iron-based systems through the careful selection of water-soluble metal precursors and modulators, often yielding products with surface areas and crystallinities comparable to those obtained from conventional solvent-based methods. For COFs, aqueous or aqueous-organic biphasic interfacial synthesis has emerged as a promising route to produce crystalline frameworks under mild conditions [57]. Room-temperature synthesis protocols have been developed for selected MOFs (particularly ZIFs and certain MIL systems) and COFs (via interfacial polymerization or acetic acid-catalyzed condensation at ambient temperature), dramatically reducing energy consumption while shortening reaction times from days to hours. Mechanochemical synthesis—performed via grinding or ball-milling of solid precursors—eliminates the need for bulk solvents entirely, offering a nearly solvent-free route that is inherently scalable and produces minimal chemical waste [31]. Electrochemical synthesis, already noted for MOFs, offers continuous production capabilities and reduced energy demands [30].

**Figure 1:**
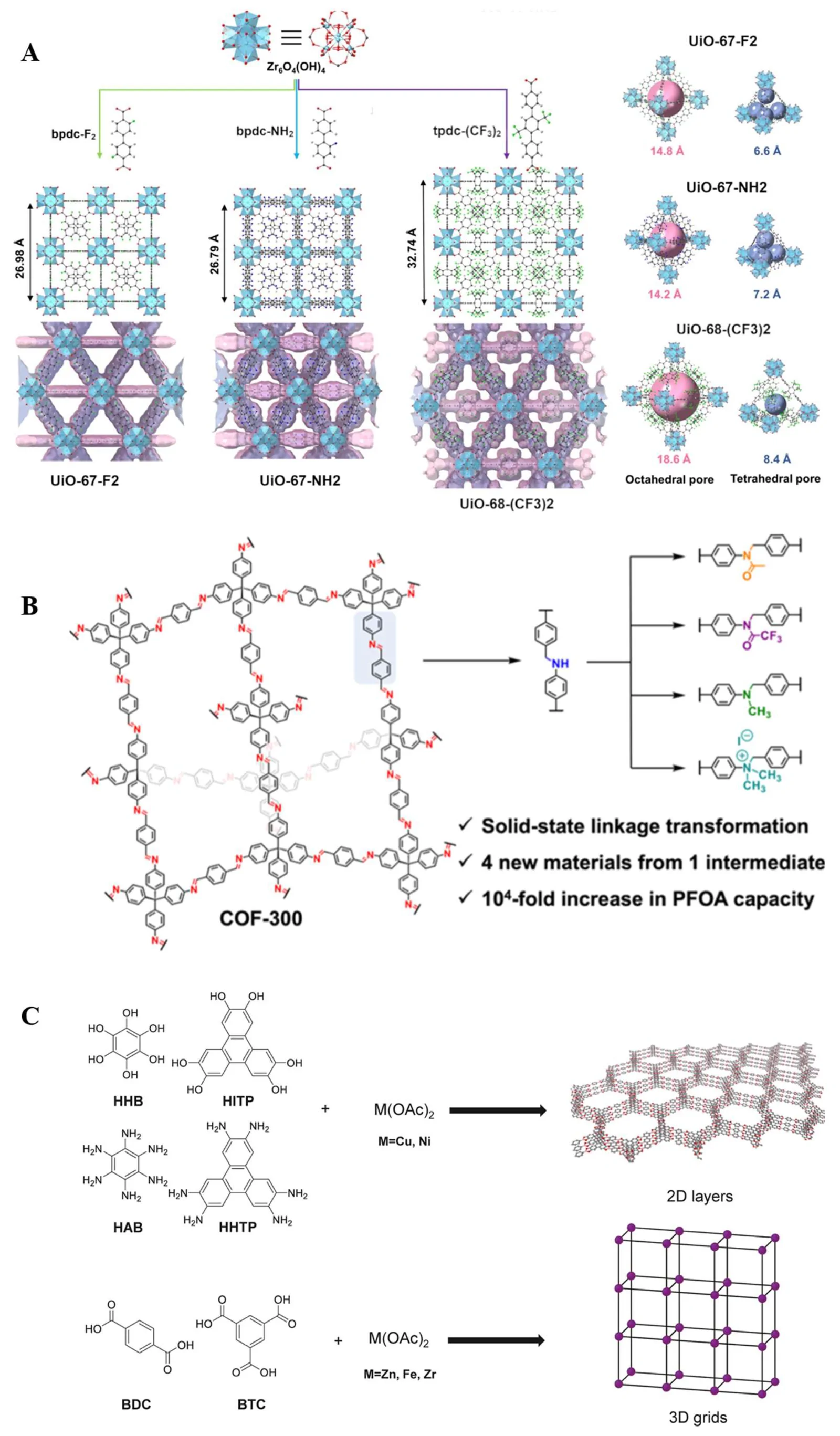
Schematic illustration of the chemical structures and primary synthesis strategies for (**A**) MOF UiO-66 series [43], (**B**) COF-300 series [62], and (**C**) MCOFs [80].

As shown in Figure 1, the three framework families share a common reticular chemistry origin but diverge in their node-linker connectivity, which fundamentally governs their respective stability, porosity, and post-synthetic modifiability. The scalability of these green approaches must be evaluated in conjunction with the product quality metrics—specific surface area, crystallinity, particle morphology, and adsorption capacity—to ensure that the synthesized materials meet the performance benchmarks established by laboratory-scale counterparts. Furthermore, the cost and availability of organic linkers, metal salts, and catalysts must be factored into the overall assessment of practical viability [78]. For COFs and MCOFs, the relatively high cost and limited commercial availability of functionalized organic building blocks currently present a bottleneck to large-scale implementation, underscoring the need for developments in efficient, low-cost synthetic routes for key monomers. Collectively, the advancement of green and scalable synthesis protocols will be instrumental in bridging the gap between the demonstrated laboratory potential of MOF, COF, and MCOF adsorbents and their practical deployment in PFAS-contaminated water treatment infrastructure.

### 2.5. Key Physicochemical Characterization and Surface Properties

The thorough physicochemical characterization of framework materials is indispensable for establishing robust structure–performance correlations and validating successful synthesis. This section summarizes the key characterization techniques employed to interrogate structural, morphological, textural, and surface chemical features relevant to PFAS adsorption [49].

**Figure 2:**
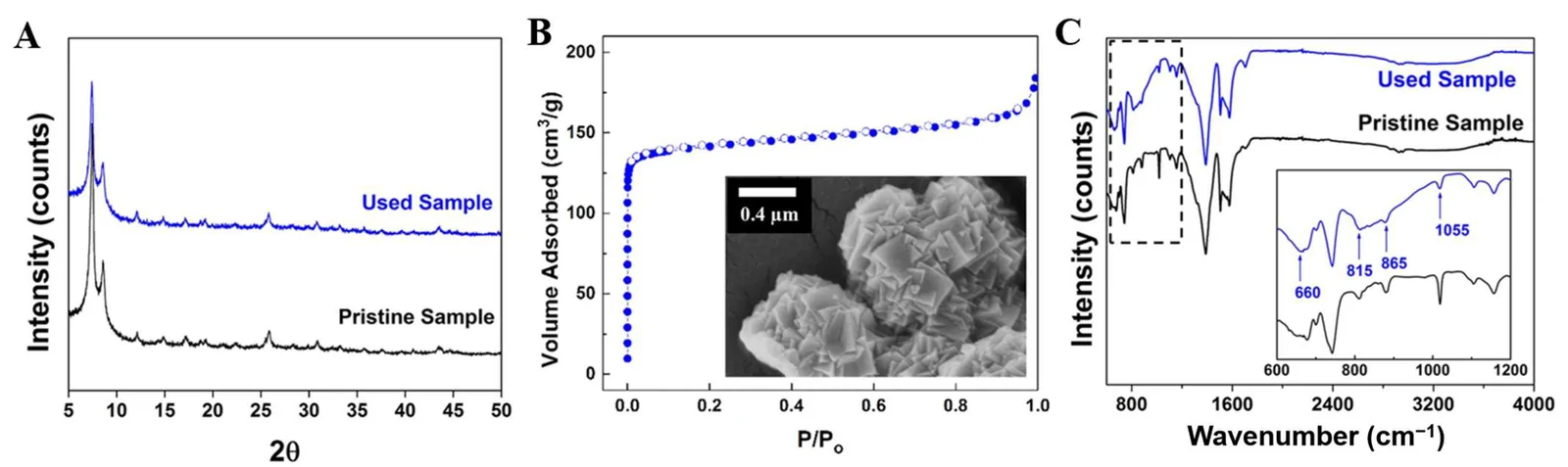
Representative characterization profiles of framework materials. (**A**) XRD [37]; (**B**) Nitrogen physisorption [37]; (**C**) FT-IR [62].

Powder X-ray diffraction (PXRD) is the foremost technique for assessing crystallinity and phase purity. For MOFs, characteristic low-angle peaks confirm long-range order, while the preservation of these peaks after aqueous exposure—particularly under varying pH conditions—provides primary evidence for hydrolytic stability (Figure 2A). For COFs and MCOFs, PXRD reveals both in-plane periodicities and interlayer stacking distances. Fourier transform infrared spectroscopy (FT-IR) confirms chemical bonding through characteristic vibrational bands (Figure 2C) [62]. Solid-state NMR complements FT-IR by providing direct evidence of covalent bond formation, such as imine (C=N) linkages confirmed by ^13^C CP-MAS signals at ~150–160 ppm [51]. Scanning and transmission electron microscopy (SEM/TEM) enable direct visualization of morphology, particle size distribution, and structural defects—including missing-linker vacancies in MOFs and layer dislocations in COFs—which profoundly influence active site accessibility and adsorption capacity [47].

Nitrogen physisorption at 77 K provides adsorption isotherms from which apparent specific surface areas can be estimated using models such as BET or Langmuir, together with model-derived pore-volume and pore-size distributions [48]. These values depend on the probe gas, sample activation conditions, selected pressure range, and underlying model assumptions; they should therefore be interpreted as operational descriptors rather than direct measurements of the true geometric surface area (Figure 2B). The relationship between pore dimensions and PFAS molecular size is critical: the molecular dynamics diameter of PFOA is ~6.2 Å, that of PFOS ~7.8 Å, while short-chain PFBA and PFBS are ~4.5 and ~5.2 Å, respectively. Materials with pore apertures smaller than the PFAS diameter impose size-exclusion effects, limiting adsorption to external surfaces (e.g., ZIF-8 with ~3.4 Å windows). Conversely, excessively large pores (>20 Å) reduce confinement effects and weaken host–guest interactions. The optimal pore regime for PFAS adsorption typically lies at 8–20 Å, allowing efficient molecular ingress while maintaining sufficient confinement.

X-ray photoelectron spectroscopy (XPS) interrogates surface elemental composition and chemical states. For PFAS-loaded adsorbents, the appearance of an F 1s peak at ~688–689 eV, accompanied by a CF_2_ component in the C 1s spectrum at ~291–292 eV, provides unequivocal evidence of PFAS uptake [50]. Binding energy shifts in the F 1s peak relative to pristine PFAS can reveal the nature of adsorbate–adsorbent interactions. Zeta potential measurements as a function of pH determine the point of zero charge (pH_p_zc), which governs electrostatic interactions with anionic PFAS. When solution pH is below pH_p_zc, the positively charged surface attracts anionic PFAS; above pH_p_zc, electrostatic repulsion suppresses adsorption. Water contact angle (WCA) measurements quantitatively indicate surface hydrophobicity, directly influencing hydrophobic and fluorophilic interactions. Hydrophobic materials with high WCA (>90°) exhibit enhanced affinity toward PFAS fluorinated tails, while hydrophilic surfaces may exhibit stronger water binding that competes with PFAS adsorption [52].

## 3. Adsorption Performance and Influencing Parameters

### 3.1. Equilibrium Isotherm and Kinetic Modeling

Isotherm Models. Equilibrium adsorption is commonly evaluated using the Langmuir and Freundlich models. The former assumes monolayer adsorption on equivalent sites, whereas the latter empirically accounts for surface heterogeneity [37,71]. For many PFAS–framework systems, the Langmuir model provides a better fit and yields maximum capacity (*q*_m_) values for performance comparison [18].

Notable examples of maximum adsorption capacities reported in the literature demonstrate the remarkable potential of these materials. For MOFs, a fluorinated UiO-67 analog, UiO-67-F2, exhibited a significantly higher PFOA adsorption capacity (Langmuir maximum *q*_m_ = 3060 mg/g) compared to unmodified UiO-67 (1589 mg/g), representing one of the highest reported values for MOF-based PFOA adsorbents [43]. For COFs, a chitosan-coated COF composite (COF@CS) achieved a maximum PFOA capacity of 2.8 mmol/g (approximately 1159 mg/g) at pH 5, with adsorption rates reaching 6.2 mmol/(g·h) [59].

For MCOFs, porphyrin-based frameworks with regulated metal centers represent the most promising development. Through a metal center engineering strategy for porphyrin-based COFs, a clear correlation was established: a narrower energy gap between the d-band center of the metal center and the Fermi level corresponds to significantly enhanced PFOA adsorption efficiency. Ce-COF exhibited an exceptional maximum adsorption capacity for PFOA of 1494.1 ± 52.0 mg/g and demonstrated high removal efficiency across a broad concentration range, from trace levels (1 μg/L, 96%) to high concentrations (20 mg/L, 99%) [81].

Kinetic modeling provides insights into the adsorption rate and mechanism. The pseudo-second-order (PSO) model, expressed as:

(1) $$\frac{t}{q_{t}}=\frac{1}{k_{2}q_{e}^{2}}+\frac{t}{q_{e}}$$

where *k*_2_ is the PSO rate constant. The PSO model is most frequently employed and generally provides excellent fits for PFAS adsorption onto framework materials, indicating that chemisorption or strong electrostatic interactions govern the rate-limiting step. The pseudo-first-order (PFO) model, ln(*q_e_* − *q_t_*) = ln*q_e_* − *k*_1_ *t*, is occasionally applicable but typically yields inferior correlations [82]. The intraparticle diffusion model, *qt* = *k_id_ t*^1/2^ + *C*, where *k_id_* is the intraparticle diffusion rate constant, is used to distinguish between boundary-layer diffusion and intraparticle diffusion as the rate-controlling step. For MOFs and COFs with well-defined pore architectures, adsorption kinetics are often rapid, with equilibrium achieved within minutes to a few hours.

The integration of isotherm and kinetic analyses provides a comprehensive framework for evaluating adsorbent performance. The Langmuir-derived *q*_m_ values enable direct comparison of maximum adsorption capacities across different materials, while kinetic parameters inform reactor design and contact time requirements. A summary of representative *q*_m_ values for MOF and COF adsorbents is presented in Table 2 and Table 3.

### 3.2. Effect of Solution Chemistry

**Figure 3:**
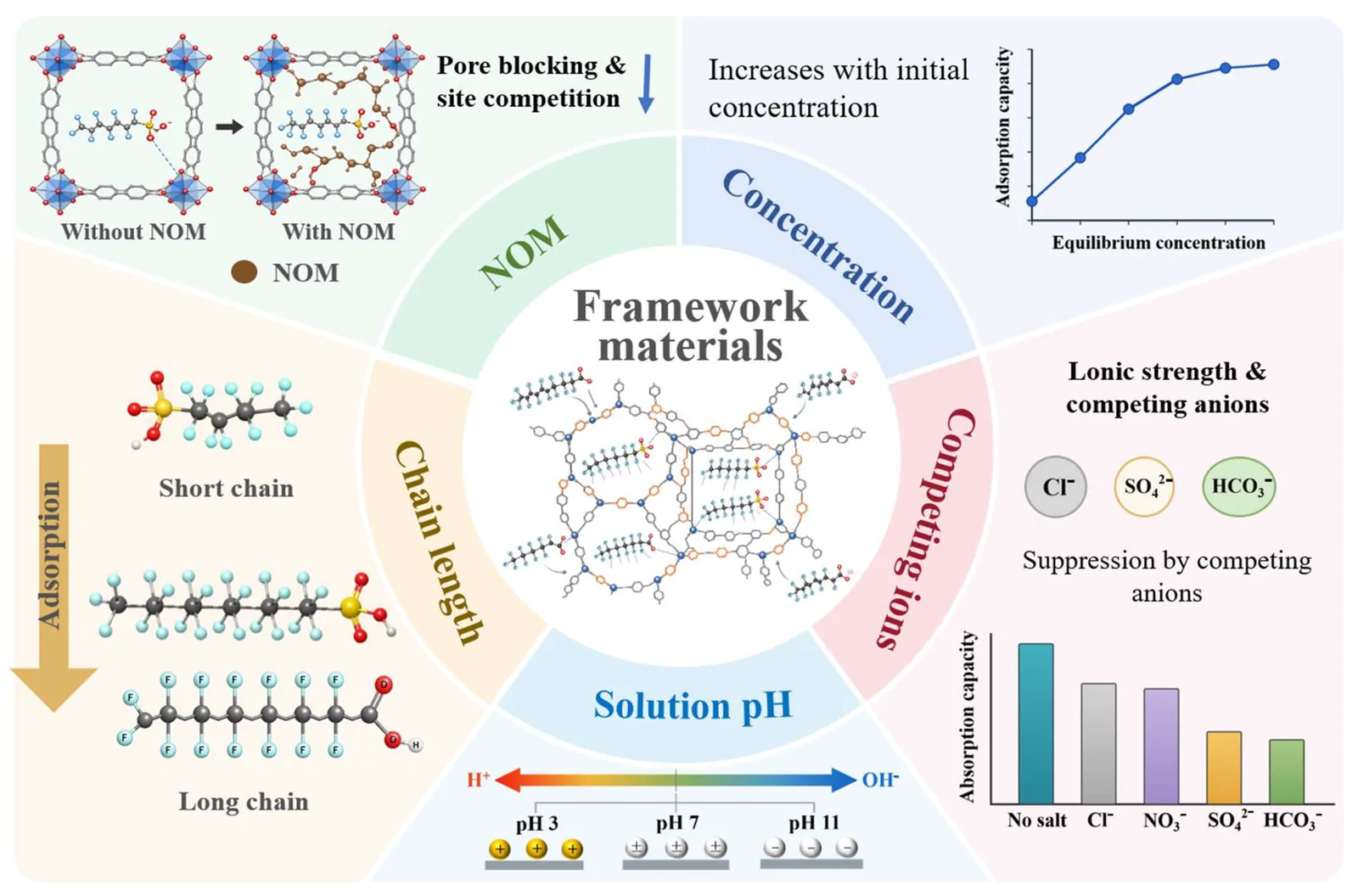
Multi-factor effects of solution conditions on adsorption performance.

The adsorption performance of framework materials toward PFAS is profoundly influenced by solution chemistry parameters, including pH, ionic strength, and the presence of coexisting natural organic matter (NOM) and competing anions (Figure 3). Understanding these effects is essential for predicting adsorbent performance in real water matrices and for optimizing operational conditions [32].

Solution pH exerts a dual influence on PFAS adsorption: it governs both the speciation and charge state of PFAS molecules and the surface charge characteristics of the adsorbent. PFAS exist predominantly as anions across environmentally relevant pH ranges (pH 5–9) due to their low pKa values—PFCAs have pKa values of approximately 2.8–3.0, while PFSAs exhibit pKa values below 1, rendering them fully deprotonated under most natural water conditions [1]. The surface charge of framework materials, quantified by zeta potential measurements, varies with pH, and the point of zero charge (pH_p_zc) marks the pH at which the net surface charge is neutral. When the solution pH is below the pH_p_zc, the adsorbent surface carries a net positive charge, promoting electrostatic attraction toward anionic PFAS species. Conversely, when the pH exceeds the pH_p_zc, the surface becomes negatively charged, resulting in electrostatic repulsion that suppresses PFAS uptake [18].

For most MOFs and COFs, PFAS adsorption capacity decreases with increasing pH. For example, UiO-66-based materials have demonstrated enhanced PFOS adsorption at pH 3 relative to pH 5 [37]. This pH-dependent behavior is attributed to the progressive deprotonation of surface hydroxyl groups and the increasing negative surface charge at higher pH values, which intensifies electrostatic repulsion with anionic PFAS. However, the magnitude of pH effects varies considerably among framework materials depending on their pH_p_zc values and the dominant adsorption mechanisms. Materials for which hydrophobic and fluorophilic interactions are the primary driving forces exhibit less pronounced pH dependence than those relying predominantly on electrostatic attraction [1]. For COFs bearing pH-responsive functional groups such as –NH_2_ (which can be protonated at low pH), the pH dependence can be particularly strong. The optimal pH for PFAS adsorption typically lies in the acidic to neutral range (pH 3–7), though the specific optimum depends on the framework’s chemical composition and the target PFAS species [18].

Ionic strength and the presence of competing anions significantly influence PFAS adsorption through multiple mechanisms. Increased ionic strength compresses the electrical double layer, reducing electrostatic repulsion between negatively charged PFAS molecules and facilitating their aggregation, which can enhance adsorption in some cases. However, high ionic strength also introduces competing anions (e.g., Cl^−^, SO_4_^2−^, HCO_3_^−^, NO_3_^−^) that can competitively occupy adsorption sites, particularly those governed by electrostatic attraction or anion exchange [83].

The nature and concentration of competing anions determine the extent of interference. Monovalent anions such as Cl^−^ and NO_3_^−^ generally exert weaker competition than divalent anions such as SO_4_^2−^ [18]. For MIL-101(Fe)-NH_2_@MgCo-LDH composites, >95% removal was sustained in the presence of Cl^−^ and NO_3_^−^, whereas stronger competition from F^−^ and SO_4_^2−^ suppressed adsorption [84]. This differential behavior reflects the higher charge density and stronger electrostatic interactions of divalent anions with positively charged surface sites. For PFOS removal, studies have shown that increasing ionic strength from 0 to 100 mM can reduce removal efficiency by approximately 20% [18]. The presence of Ca^2+^ and Mg^2+^ cations can also influence adsorption through bridging effects or by altering the PFAS speciation. In general, framework materials that rely predominantly on hydrophobic and fluorophilic interactions—rather than electrostatic forces—exhibit greater resistance to ionic strength interference.

The presence of NOM in natural waters poses a significant challenge to PFAS adsorption. NOM molecules, which include humic and fulvic acids, can interfere through multiple mechanisms: (1) competitive adsorption for binding sites on the framework surface; (2) pore-blocking effects that restrict PFAS access to internal porosity; (3) formation of NOM–PFAS complexes that alter PFAS speciation and bioavailability; and (4) surface fouling that reduces the effective surface area and active site density.

Studies have consistently demonstrated that increasing NOM concentrations lead to reduced PFAS adsorption capacities. The extent of NOM interference depends on the NOM concentration, molecular weight distribution, and chemical composition, as well as the framework material’s surface properties. Hydrophobic NOM fractions tend to compete more strongly for hydrophobic adsorption sites, while hydrophilic NOM fractions may have less impact. The pore-blocking effect is particularly relevant for microporous frameworks with narrow pore windows, where NOM molecules can physically obstruct pore entrances and prevent PFAS ingress. Mesoporous frameworks with larger pore apertures generally exhibit greater resistance to pore-blocking [18]. For practical water treatment applications, the performance of framework adsorbents must be evaluated in real water matrices containing background NOM to assess their true remediation potential [21,84].

### 3.3. Chain Length and Functional Group Specificity

The molecular structure of PFAS—particularly the perfluoroalkyl chain length and the terminal functional head group—exerts a decisive influence on adsorption affinity, capacity, and selectivity toward framework materials. Understanding these structure–performance relationships is essential for designing adsorbents capable of addressing the full spectrum of PFAS contaminants.

Long-chain PFAS, exemplified by PFOA (C8) and PFOS (C8), generally exhibit substantially higher adsorption affinities and capacities on framework materials compared to their short-chain counterparts such as PFBA (C4) and PFBS (C4) [18,23]. This chain-length-dependent behavior arises from several interconnected factors. First, the hydrophobic fluorinated tail becomes increasingly hydrophobic with increasing chain length, as reflected by the higher log Kow values—PFOA has a log Kow of 4.81 while PFBA has only 2.82 [1]. This enhanced hydrophobicity drives stronger hydrophobic interactions with the framework surfaces and promotes partitioning from the aqueous phase into the adsorbent’s hydrophobic domains. Second, fluorophilic (F–F) interactions between the perfluorinated alkyl chains and fluorine-containing functional groups on the framework surface intensify with longer chains due to the greater number of C–F bonds available for dispersion forces. Third, longer-chain PFAS exhibit stronger van der Waals interactions with pore walls, increasing the overall adsorption energy.

Experimental data consistently confirm this trend. For MIL-101(Fe)-NH_2_@MgCo-LDH composites, adsorption efficiency increased progressively with perfluoroalkyl chain length from C4 (82.5%) to C9 (98.9%) [84]. Similarly, NH_2_-functionalized MOFs demonstrated adsorption efficiency that increased with chain length from C4 to C9 [1]. For COFs, computational studies have revealed that when a PFAS molecule is within the 8.0 Å pore of CTF-1, the potential well depths are -10 kcal/mol for PFBA, −24 kcal/mol for PFOA, and −28 kcal/mol for PFOS, indicating significantly stronger thermodynamic driving forces for long-chain PFAS adsorption.

The practical implication of this chain-length dependence is that short-chain PFAS—which are increasingly used as replacements for regulated long-chain compounds—pose a more formidable challenge for adsorption-based remediation. Their higher water solubility, lower hydrophobicity, and weaker affinity for hydrophobic surfaces render them more difficult to remove using conventional adsorbents [18,25]. Framework materials designed specifically for short-chain PFAS capture require tailored pore sizes (typically 5–8 Å to provide adequate confinement) and enhanced surface functionality to compensate for the reduced hydrophobic driving force.

Molecular dynamics simulations have revealed that kinetic factors favor the adsorption of short-chain PFAS due to faster diffusion into nanopores, even though larger PFAS are thermodynamically favored. This kinetic advantage offers a potential pathway for designing selective short-chain PFAS adsorbents.

The global transition away from legacy long-chain PFAS has led to the proliferation of alternative compounds, including GenX (HFPO-DA, hexafluoropropylene oxide dimer acid), F-53B (a chlorinated polyfluoroalkyl ether sulfonate), and various HFPO oligomers [85]. These emerging alternatives present distinct structural features—ether linkages, branched architectures, and in the case of F-53B, chlorine substituents—that influence their adsorption behavior on framework materials.

GenX adsorption has been investigated on several COF platforms. Imine-linked two-dimensional COFs bearing primary amines adsorb GenX rapidly at environmentally relevant concentrations, with the synergistic combination of polar amine groups and hydrophobic surfaces responsible for GenX binding [63]. Fluorinated amino COFs (COF-FS-NH_2_) exhibited maximum adsorption capacities of 0.210 mmol/g for GenX, 0.319 mmol/g for C7 HFPO-TA, and 0.289 mmol/g for HFPO-TA, as determined by the Langmuir model [66]. Cationic COFs with quaternary ammonium groups have shown high adsorption capacities for GenX (2.06 mmol/g) and HFPO-TA (2.16 mmol/g), outperforming conventional activated carbons and resins [86]. The branched structure and ether oxygen of GenX introduce additional polar interactions—particularly hydrogen bonding with surface functional groups—that can complement hydrophobic interactions, potentially enhancing adsorption on appropriately functionalized frameworks.

For F-53B and other perfluoroalkyl ether sulfonic acids, the presence of the ether linkage introduces conformational flexibility and additional hydrogen-bonding acceptor sites. The chlorine substituent in F-53B adds a unique polarizable moiety that may engage in halogen bonding or enhanced van der Waals interactions with framework surfaces. However, systematic studies of these emerging alternatives on MOF, COF, and MCOF platforms remain limited, representing a significant research gap. The development of framework materials capable of efficiently removing both legacy and emerging PFAS—including the increasingly prevalent short-chain and ether-containing alternatives—requires continued investigation into the molecular-level interactions governing adsorption selectivity and affinity across the diverse PFAS chemical space.

## 4. Mechanistic Elucidation of PFAS-Framework Interactions

**Figure 4:**
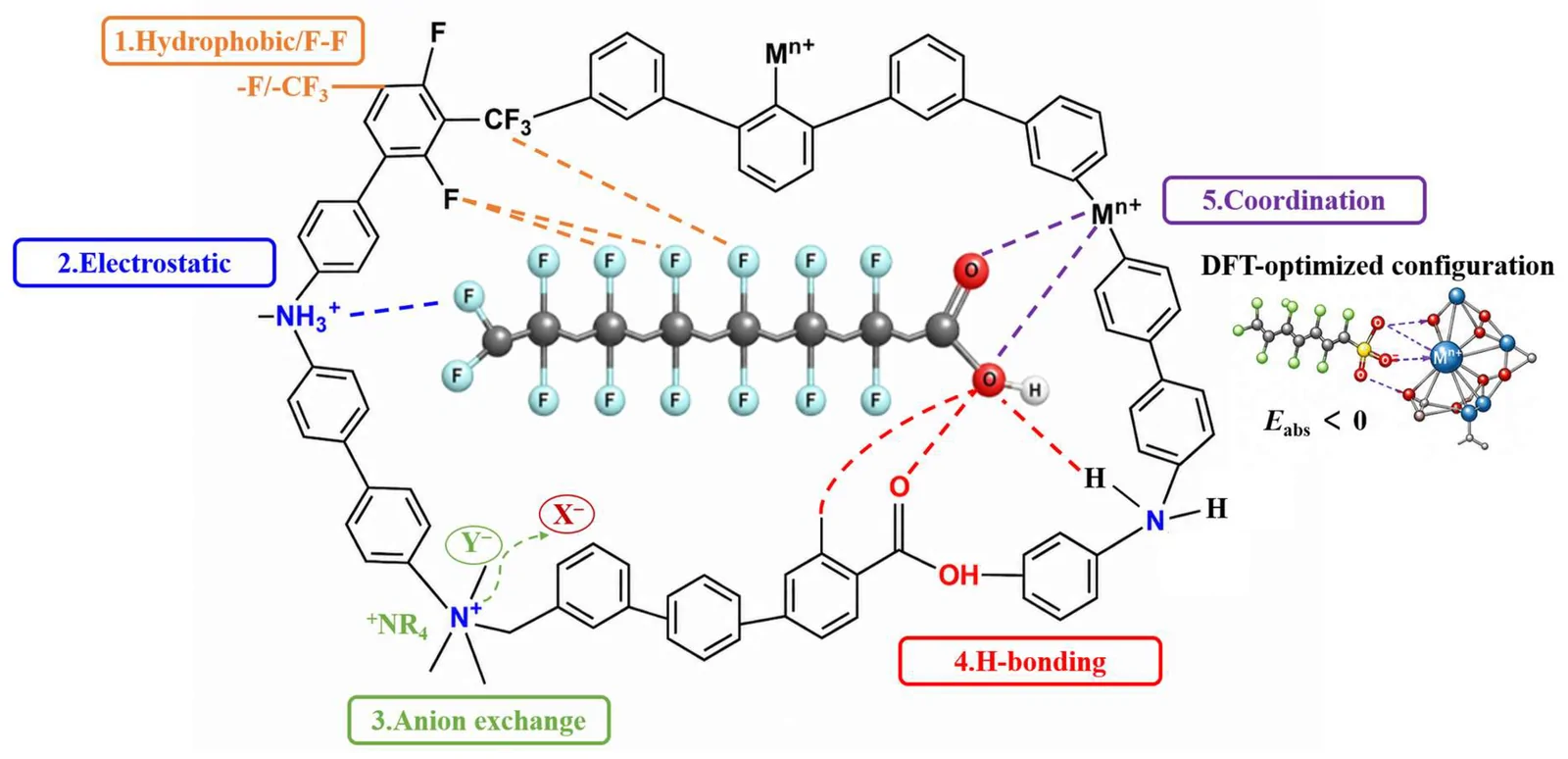
Schematic illustration of multiple adsorption mechanisms of PFAS within the pores of framework materials.

PFAS adsorption within framework pores should be regarded as a coupled transfer process rather than as five independent interactions (Figure 4). Its overall thermodynamic driving force is the adsorption free energy. Electrostatic attraction, hydrogen bonding, anion exchange, and coordination primarily stabilize the ionic head group, whereas hydrophobic/fluorophilic effects and dispersion contacts act mainly on the perfluoroalkyl tail. Although these mechanisms cannot be assigned exclusively to enthalpy or entropy, direct host–guest interactions largely determine the interaction energy, while desolvation, counterion and water release, conformational restriction, and pore-water reorganization introduce additional enthalpic and entropic contributions. Their relative importance therefore depends on the PFAS head group and chain length, solution pH and ionic strength, and framework pore size, wettability, and site accessibility [32,87,88,89,90,91].

### 4.1. Multiple Interaction Pathways

The hydrophobic fluorinated alkyl tails of PFAS molecules drive their partitioning from the aqueous phase onto the hydrophobic surfaces of framework materials [18]. For long-chain PFAS such as PFOA and PFOS, this hydrophobic effect is particularly pronounced, as reflected by their high log Kow values (4.81 and 6.28, respectively) [1]. In fluorine-functionalized frameworks, an additional fluorophilic (F–F) interaction arises from the favorable dispersion forces between perfluorinated alkyl chains and fluorine-containing functional groups on the pore walls. DFT calculations on fluorinated MOFs have revealed that the incorporation of –CF_3_ groups significantly enhances the adsorption energy of PFOA through synergistic F–F interactions [87]. A bifunctional Zr-MOF (MOF-808-TFMA) grafted with trifluoromethylalanine moieties demonstrated exceptional PFOA and PFBA capacities of 1558.3 and 605.4 mg/g, respectively, with DFT calculations confirming that fluorophilic and hydrophobic interactions preferentially enhance long-chain PFAS capture [88].

PFAS exist predominantly as anions across environmentally relevant pH ranges due to their low pKa values—PFCAs have pKa values of approximately 2.8–3.0, while PFSAs exhibit pKa values below 1 [1]. Positively charged framework surfaces therefore exert strong electrostatic attraction toward anionic PFAS species. Zeta potential measurements provide direct evidence of this charge-mediated mechanism: for the core–shell UiO-66-NH_2_-on-MIL-101(Cr) composite, a significant zeta potential change (Δζ = 36.1 mV) was observed upon PFOA adsorption, confirming electrostatic attraction as a contributing mechanism [89]. Anionic-exchange MOFs, such as MIL-101(Cr) modified via post-synthetic modification (PSM), exhibited PFOA adsorption capacities of 1.89 mmol/g, with experimental and DFT results revealing that anion exchange operates in conjunction with Lewis acid/base complexation and electrostatic interactions [41]. For COFs, functionalization with amino groups introduces positive electrostatic potential within the pores—COF–NH_2_ exhibited a positive potential of 12.85 kcal/mol, which preferentially attracted anionic PFAS and resulted in a surface-to-pore conversion rate of approximately 75%, compared to only 40% for cyano-functionalized COF–CN with a negative potential of −30 kcal/mol [90]. Cationic COFs bearing quaternary ammonium groups have further demonstrated high adsorption capacities for GenX (2.06 mmol/g) and HFPO-TA (2.16 mmol/g) through enhanced anion-exchange interactions [86].

Perhaps the most distinctive adsorption mechanism for MOFs and MCOFs involves the direct coordination of PFAS head groups—particularly carboxylate (–COO^−^) and sulfonate (–SO_3_^−^) moieties—to unsaturated metal sites or metal clusters. XPS provides critical evidence for this coordination mechanism: upon PFOA adsorption onto Fe-based MOFs, a decrease in the binding energy of Fe 2p and an increase in the binding energy of F 1s were observed, confirming the presence of Lewis acid/base complexation between the metal centers (Lewis acids) and the PFOA carboxylate groups (Lewis bases) [91]. For the UiO-66-NH_2_-on-MIL-101(Cr) composite, DFT calculations combined with XPS and FT-IR analysis revealed that the primary adsorption mechanism involves strong coordination interactions between PFOA carboxylate groups and metal clusters (Cr/Zr–O), with binding energies of E_b_ = −178.02 kcal/mol for Cr–O and −121.72 kcal/mol for Zr–O. These strong coordination interactions are supplemented by hydrogen bonding (O^−^⋯H–N/H–O), π-CF hydrophobic interactions, and electrostatic attraction, collectively contributing to the remarkable *q*_m_ of 860 mg/g [89]. FT-IR analysis of PFBS adsorption on NU-1000 further confirmed the involvement of metal–anion interactions through characteristic spectral shifts [35]. PFAS adsorption arises from five coupled contributions rather than independent mechanisms, as summarized in Figure 4. Their relative importance depends on PFAS structure and adsorption conditions. Hydrophobic and dispersion interactions contribute more strongly to long-chain PFAS adsorption, whereas electrostatic attraction, anion exchange, hydrogen bonding, and coordination become relatively important for short-chain PFAS [88]. MOFs can provide metal nodes or open metal sites for head-group coordination, whereas COFs rely mainly on charged or polar linkers, hydrophobic pore walls, and pore confinement [63,86,89,90]. Thus, adsorption in both material classes reflects the overall free-energy balance among direct binding, desolvation, and pore accessibility rather than a single mechanism.

### 4.2. Contribution of Metal Centers in MCOFs

MCOFs offer a unique platform for PFAS adsorption by integrating coordinatively bound metal centers within covalently bonded organic backbones [81]. Unlike MOFs, where metal clusters serve as integral nodal components and are often sterically crowded, MCOFs feature metal ions anchored to well-defined chelating sites—such as porphyrin cores, bipyridine moieties, or imine nitrogens—within the organic struts [75]. This architectural distinction confers two critical advantages: (1) the metal centers in MCOFs are spatially isolated and more accessible to guest molecules, and (2) the coordination environment of the metal can be systematically tuned through the choice of chelating ligand and metal ion, enabling precise control over adsorption energetics.

The importance of metal center selection in MCOFs has been systematically elucidated through a metal center engineering strategy for porphyrin-based COFs. Leveraging DFT calculations and experimental analysis across five metals (Ti, Fe, Cu, In, Ce), a clear correlation was established: a narrower energy gap between the d-band center of the metal center and the Fermi level corresponds to significantly enhanced PFOA adsorption efficiency [81]. Among the metals investigated, Ce-COF exhibited the most favorable d-band center alignment and demonstrated an exceptional maximum adsorption capacity for PFOA of 1494.1 ± 52.0 mg/g, with high removal efficiency across a broad concentration range—from trace levels (1 μg/L, 96%) to high concentrations (20 mg/L, 99%) [81]. This d-band center descriptor provides a powerful predictive tool for the rational design of MCOF adsorbents, as it directly correlates the electronic structure of the metal center with the strength of metal–PFAS interactions.

Comparing MOFs and MCOFs from a mechanistic perspective reveals fundamental differences in the nature of metal–PFAS binding. In MOFs, PFAS molecules coordinate to metal clusters that are integral to the framework nodes, often through bridging or bidentate binding modes involving multiple metal centers [41,89]. In MCOFs, by contrast, the metal centers are anchored to the organic backbone via chelation and present a more defined and tunable coordination microenvironment [75]. DFT calculations on MCOF systems have demonstrated that the adsorption energy of PFOA is strongly correlated with the d-band center position of the metal, with metals exhibiting higher d-band occupancy (closer to the Fermi level) showing stronger binding affinities. This electronic-level understanding suggests that MCOFs can achieve metal–anion interaction strengths comparable to or even exceeding those of MOFs, while benefiting from the superior chemical stability of the covalent COF backbone. The ability to independently tune the metal center (through metal ion selection) and the organic backbone (through monomer design) makes MCOFs a highly versatile platform for optimizing both the thermodynamic driving forces and the structural stability of PFAS adsorbents.

### 4.3. Theoretical Quantification of Coupled Adsorption Driving Forces

DFT and molecular dynamics (MD) provide complementary connections between the five contributions in Figure 4 and the thermodynamic framework described above. DFT-derived adsorption energies, charge density differences, and density-of-states analyses reveal the electronic origin and site-specific energetic stabilization of coordination, electrostatic, hydrogen-bonding, and dispersion interactions [41,89,91,92,93]. However, static adsorption energies (*E_ads_*) are not equivalent to aqueous adsorption free energies (*ΔG_ads_*), because solvation and entropy are generally omitted or approximated. MD simulations resolve hydration, pore confinement, molecular motion, and diffusion and, when combined with free-energy sampling, can assess solvent-mediated and entropic contributions [90,94,95]. Reported *E_ads_* values should therefore be interpreted as site-level binding descriptors, whereas overall adsorption favorability reflects the combined effects of direct binding, desolvation, and confinement.

Adsorption energy calculations provide a direct measure of the thermodynamic favorability of PFAS–framework interactions. For the UiO-66-NH_2_-on-MIL-101(Cr) composite, DFT calculations revealed binding energies of *E_b_* = −178.02 kcal/mol for Cr–O coordination and −121.72 kcal/mol for Zr–O coordination, confirming that the Cr sites offer stronger binding than the Zr sites [89]. For MOF-808-TFMA, DFT calculations elucidated a multi-synergistic site mechanism wherein amino-enabled hydrogen-bonding and electrostatic interactions dominate short-chain PFAS capture, while fluorophilic and hydrophobic interactions preferentially enhance long-chain PFAS adsorption [88]. For MIL-101(Cr)-based anionic-exchange MOFs, DFT calculations confirmed that in addition to anion exchange, the major PFOA adsorption mechanism involves a combination of Lewis acid/base complexation between PFOA and Cr(III) and electrostatic interaction between PFOA and protonated carboxyl groups of the BDC linker [41].

Charge density difference analysis provides visualization of electron redistribution upon PFAS adsorption, revealing the nature and direction of charge transfer between the adsorbate and the framework. For Fe-based MOFs, differential charge analysis demonstrated that the Fe center and the benzene ring of organic ligands serve as the electron acceptor and donor, respectively, in the adsorption process. This electron transfer facilitates Lewis acid/base complexation, which dominates the adsorption process due to the highest overlap of electron clouds [91].

Density of states (DOS) analysis offers insights into the electronic structure changes that accompany PFAS adsorption. By comparing the partial DOS of the framework before and after PFAS uptake, researchers can identify which orbitals are primarily involved in binding and how the electronic structure is modified upon adsorption [93]. For MCOFs, the d-band center of the metal—derived from DOS analysis—has emerged as a critical descriptor for predicting adsorption performance. The d-band center model, originally developed for heterogeneous catalysis, has been successfully extended to PFAS adsorption on MCOFs: a narrower energy gap between the d-band center and the Fermi level correlates with stronger metal–PFAS interactions and higher adsorption capacities.

While DFT calculations provide static, electronic-level insights into adsorption at specific binding sites, MD simulations capture the dynamic behavior of PFAS molecules within the confined pore environments of framework materials. MD simulations reveal the conformational evolution of PFAS upon entering nanopores, the role of pore size in governing adsorption selectivity, and the influence of hydration layers on the adsorption process [94].

Pore size effects on PFAS adsorption have been systematically investigated using MD simulations on covalent triazine-based frameworks (CTFs) of varying pore diameters. This mechanistic adsorption data revealed a “goldilocks zone” in pores with diameters of approximately 8 Å, where PFAS molecules thread through the pores smoothly. Kinetic factors from diffusion into these nanopores favor the adsorption of short-chain PFAS, even though larger PFAS are thermodynamically favored. Each pore tends to initially adsorb only one PFAS molecule, occupying the mouth of the pore, until the local COF surface is saturated, after which multiple occupancy per pore can occur. These findings provide critical design principles for enhancing both selectivity and capacity in PFAS adsorbent materials.

MD simulations on functionalized COF membranes have revealed multi-stage PFAS adsorption, progressing from surface attachment to pore equilibration. The key factor governing adsorption efficiency was found to be the surface-to-pore conversion rate, which was approximately 75% for COF–NH_2_ compared to only 40% for COF–CN [90]. This difference was attributed to functional group-induced electrostatic potential within the pores—COF–CN exhibited a negative potential of approximately −30 kcal/mol due to the cyano groups, while COF–NH_2_ showed a positive potential of 12.85 kcal/mol from the amino groups [90]. PFAS, existing as anions in aqueous solution, preferentially occupied the positively charged pores of COF–NH_2_. Additionally, the dual hydration layers of COF–CN restricted PFAS access, resulting in lower adsorption, whereas the asymmetric hydration on COF–NH_2_ facilitated entry while limiting escape, thereby enhancing adsorption efficiency.

MD simulations have further elucidated the conformational changes in PFAS upon entering nanopores. Radial distribution function (RDF) analysis revealed that all three PFAS species (PFBA, PFOA, and PFOS) primarily bind with the first and second layers of the pore, with the hydrophilic head group interfacing with the aqueous phase. The simulations also demonstrated that PFAS molecules are reversibly adsorbed and desorbed during dynamic trajectories, providing insights into the kinetics of the adsorption–desorption process [95]. The integration of DFT and MD approaches thus offers a comprehensive multiscale understanding of PFAS adsorption—from the electronic-level details of binding at specific sites (DFT) to the dynamic, collective behavior of molecules within nanoporous environments (MD).

## 5. Comprehensive Performance Assessment and Application in Water Treatment Systems

**Figure 5:**
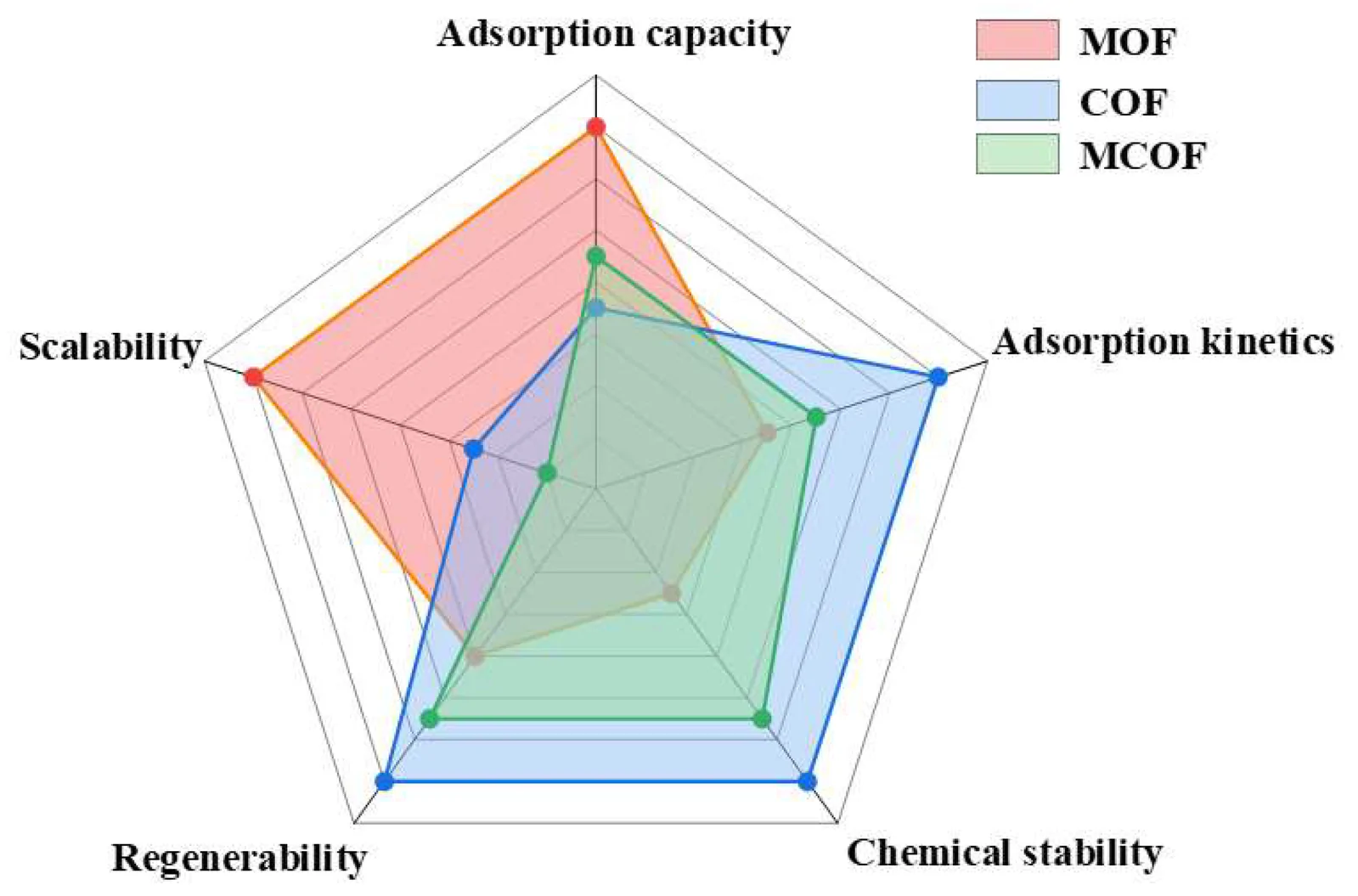
Comprehensive performance radar chart of MOF, COF, and MCOF materials.

The translation of MOF, COF, and MCOF adsorbents from laboratory research to practical water treatment requires a holistic evaluation encompassing adsorption performance, durability, regenerability, economic feasibility, and integration into real treatment systems [18,32]. This chapter provides a critical comparative assessment of the three framework families under a unified analytical framework, emphasizing direct comparisons under equivalent conditions, normalized performance metrics, and practical considerations essential for technological translation (Figure 5) [96,97].

### 5.1. Benchmarking Adsorption Capacities and Kinetics

Direct comparison of maximum adsorption capacities (*q*_m_) is complicated by variations in experimental conditions [18]. To enable meaningful cross-material comparisons, normalization of *q*_m_ by specific surface area (BET) isolates the intrinsic affinity of the material surface from total available area. Surface-area-normalized capacities (*q*_m,SA_, mg m^−2^) reveal that MCOFs exhibit significantly higher intrinsic affinity (e.g., Ce-COF: ~1.25 mg m^−2^) compared to MOFs (0.35–0.54 mg m^−2^) and COFs (0.47–0.51 mg m^−2^), attributable to synergistic contributions of metal coordination sites and covalent backbone functionality [81]. In terms of kinetics, COFs generally reach equilibrium faster (minutes to 2 h) than MOFs (2–24 h) due to their well-ordered 1D channels, while MCOFs display intermediate kinetics (1–12 h) depending on metal loading [32,98].

### 5.2. Chemical Stability and Long-Term Durability

Chemical stability under aggressive conditions is critical for practical application. β-Ketoenamine-linked COFs exhibit superior stability across a broad pH range (2–12) due to irreversible covalent linkages, outperforming MOFs (pH 4–9) which can degrade under strongly acidic or basic conditions due to hydrolysis of coordination bonds [47]. MCOFs demonstrate intermediate stability (pH 3–11), benefiting from the covalent backbone while the metal coordination sites may be susceptible to demetalation under extreme conditions [81]. Quantitative assessment via crystallinity retention index (CRI) from PXRD analysis confirms this trend: COFs retain >95% crystallinity after 24 h at pH 3, compared to 85% for MOFs and 92% for MCOFs. Thermal stability follows a similar hierarchy: COFs (350–500 °C) > MCOFs (300–450 °C) > MOFs (250–400 °C) [47].

### 5.3. Regeneration Strategies and Reusability Cycles

Three regeneration strategies have been explored: solvent desorption (methanol/NaCl), thermal regeneration, and photocatalytic in situ degradation [95,99]. Solvent desorption is most widely employed, with COFs maintaining >90% capacity over 5–10 cycles due to superior chemical stability [59], compared to 80–95% over 3–5 cycles for MOFs and 85–90% over 4–8 cycles for MCOFs. Thermal regeneration (300–500 °C) is suitable for COFs and MCOFs but causes framework collapse in many MOFs above 300 °C [47]. Photocatalytic in situ degradation offers an attractive solvent-free alternative, though mineralization efficiency remains challenging. A comprehensive reusability assessment incorporating capacity retention, structural integrity, regeneration efficiency, and cycle number reveals that COFs excel in stability and cycle life, MOFs offer ease of synthesis and high initial capacity, while MCOFs provide a balanced profile with promising durability [81].

### 5.4. Practicality and Cost Feasibility

Economic feasibility for high-volume PFAS treatment should be evaluated using the cost per unit volume of treated water rather than batch-derived maximum adsorption capacity alone. A techno-economic analysis of four representative MOFs projected production costs of US$35–71/kg for conventional solvothermal synthesis and US$13–36/kg for aqueous synthesis or liquid-assisted grinding at a hypothetical production scale of 2.5 million kg/year [100]. These figures represent projected manufacturing costs rather than PFAS treatment costs. For comparison, a full-scale GAC system reported costs of €0.020–0.10/m^3^ of PFAS-contaminated water, depending on the treatment target and media service life [101]. Because comparable cost-per-volume data are unavailable for the framework adsorbents evaluated here, GAC and ion-exchange resins currently remain more practical for high-volume treatment. Among the three framework classes, aqueous-synthesized MOFs prepared from relatively inexpensive precursors offer the most credible cost-reduction pathway [78,100], whereas COFs require specially synthesized organic monomers [47,48] and MCOFs involve additional metalation steps [74,75]. COFs and MCOFs may therefore be more appropriate for polishing or point-of-use treatment until their fixed-bed lifetime, regeneration requirements, and treatment costs have been established.

### 5.5. Batch Adsorption Versus Dynamic Column Operations

While batch experiments provide fundamental parameters, fixed-bed column tests more accurately simulate real-world conditions. Breakthrough curves—plots of effluent concentration versus throughput volume—characterize column performance and are modeled using the Thomas or Yoon–Nelson models. Column studies reveal that breakthrough time and capacity depend on adsorbent particle size, bed height, flow rate, and influent concentration. Smaller particles offer faster mass transfer but increase pressure drop [18]. The breakthrough curve shape provides insights into mass transfer resistance: sharper curves indicate favorable kinetics and efficient pore diffusion, while broader curves suggest mass transfer limitations. COFs and MCOFs with well-defined pore architectures generally exhibit sharper breakthrough curves due to more uniform pore structures compared to MOFs.

### 5.6. Competitive Adsorption in Real Water Matrices

Competitive adsorption in real water is governed by both adsorption affinity and solute abundance. Consequently, background ions and NOM present at concentrations far above those of PFAS may occupy adsorption sites even when their intrinsic affinity is lower. Consequently, abundant background solutes may occupy adsorption sites even when their intrinsic affinity is lower than that of PFAS. Cl^−^ and NO_3_^−^ often exert relatively weak competition, whereas SO_4_^2−^ and PO_4_^3−^ compete more strongly for cationic or open-metal sites. HCO_3_^−^ contributes through both site competition and pH buffering, while NOM causes site occupation, hydrophobic association, pore blocking, and surface fouling [37,83,84]. Because ΔG_ads_ includes direct binding, desolvation, and counterion or water release, no universal competition order applies. MOFs containing charged nodes or open metal sites are particularly susceptible to competing oxyanions. Cationic COFs are similarly affected when anion exchange dominates, whereas bifunctional COFs combining ionic and fluorinated domains can better resist ionic strength and NOM [64]. MCOFs may benefit from tunable metal–PFAS coordination and stable covalent pores, although competing oxyanions may also bind their metal centers, and comparative data remain limited [81,97]. Thus, mixed-mode binding and resistance to fouling are more reliable design criteria than adsorption capacity measured in single-solute water.

### 5.7. Composite Materials for Improved Processability

The micro/nanocrystalline nature of framework materials presents challenges for recovery and handling. Magnetic composites (Fe_3_O_4_@Framework) enable rapid adsorbent recovery using external magnetic fields, achieving complete separation within seconds while maintaining 70–90% of the original adsorption capacity [71]. Mixed-matrix membranes (MMMs) incorporating framework particles into polymeric matrices (PVDF, polysulfone) enable continuous flow-through operation with PFAS rejection efficiencies of 80–95% [102,103]. COFs and MCOFs with superior water stability are particularly suitable for membrane applications requiring long-term exposure to flowing water. Composite format selection depends on the application: magnetic composites for batch treatment of small-volume samples, membrane composites for continuous treatment of large water streams, and mixed-matrix composites for point-of-use devices [104]. The progressive development of composite materials is essential for translating the exceptional performance of framework adsorbents into deployable water treatment technologies [32,49].

## 6. Current Challenges, Research Gaps, and Future Roadmap

### 6.1. Persistent Bottlenecks

Despite remarkable progress in removing long-chain PFAS (C ≥ 6) using framework materials, the efficient abatement of ultrashort-chain PFAS (C_2_–C_4_), including trifluoroacetic acid (TFA), perfluoropropionic acid (PFPrA), and perfluorobutanoic acid (PFBA), remains a formidable challenge [18]. These compounds exhibit substantially higher water solubility, lower hydrophobicity (log Kow < 3), and weaker affinity for hydrophobic surfaces compared to their long-chain counterparts [1]. The molecular diameters of ultrashort-chain PFAS (3.5–4.5 Å) are significantly smaller than the pore apertures of most framework materials, resulting in weak confinement effects and reduced host–guest interaction energies [18,94]. Molecular dynamics simulations have revealed that while kinetic factors favor the adsorption of short-chain PFAS due to faster diffusion into nanopores, the thermodynamic driving forces are substantially weaker, with potential well depths in 8.0 Å pores of CTF-1 being −10 kcal/mol for PFBA compared to −24 kcal/mol for PFOA. Framework materials designed specifically for ultrashort-chain PFAS capture require pore sizes precisely tailored to provide adequate confinement (typically 4–6 Å) and enhanced surface functionality to compensate for the reduced hydrophobic driving force [94]. However, such narrow pores inevitably reduce accessibility for larger PFAS molecules, presenting a fundamental trade-off between broad-spectrum removal and selectivity for specific chain lengths.

The high salinity and high organic matter content typical of industrial wastewater and landfill leachate pose additional challenges. At ionic strengths exceeding 100 mM, the compression of the electrical double layer reduces electrostatic attraction, while competing anions (Cl^−^, SO_4_^2−^, HCO_3_^−^) occupy binding sites and induce adsorbent surface fouling [83]. For materials relying predominantly on electrostatic interactions—such as amino-functionalized MOFs and cationic COFs—this selective collapse can reduce PFAS removal efficiency by over 50% in real wastewater matrices [84]. The presence of high concentrations of NOM (20–100 mg/L TOC) further exacerbates performance degradation through competitive adsorption, pore blocking, and irreversible surface fouling. These matrix effects critically limit the applicability of many high-performance framework materials in real-world remediation scenarios. Future benchmarking should therefore evaluate Cl^−^, NO_3_^−^, HCO_3_^−^, SO_4_^2−^, PO_4_^3−^, and NOM under standardized single-component and mixed-matrix conditions while controlling pH and ionic strength. Fixed-bed breakthrough and regeneration tests are also required because batch capacities cannot capture progressive site occupation, displacement, and fouling.

A persistent obstacle to meaningful cross-material comparison is the absence of standardized adsorption testing protocols [32]. Literature reports exhibit wide variations in experimental conditions—initial PFAS concentrations (ranging from μg/L to mg/L), adsorbent dosages (0.01–10 g/L), solution pH (3–9), ionic strength (0–100 mM), contact time (minutes to days), and temperature (20–60 °C). These disparities render direct comparison of reported *q*_m_ values unreliable and complicate the identification of truly superior materials [18]. Furthermore, discrepancies in isotherm model selection (Langmuir vs. Freundlich vs. Sips), kinetic model fitting procedures, and the treatment of equilibrium data introduce additional uncertainties. The frequent reporting of theoretical Langmuir *q*_m_ values derived from models fitted to data acquired at unrealistically high PFAS concentrations (e.g., 100–1000 mg/L) further obscures performance at environmentally relevant trace levels (ng/L to μg/L). Consensus on standardized testing protocols—including recommended PFAS concentration ranges, background electrolyte composition, pH control, and equilibration time—is urgently needed to enable rigorous material benchmarking [32].

### 6.2. Future Directions Toward High-Performance Adsorbents

The controlled introduction of structural defects—such as missing linkers, missing clusters, and coordination vacancies—represents a powerful strategy for enhancing PFAS adsorption performance [37]. In MOFs, missing-linker defects expose additional open metal sites and increase the density of coordinatively unsaturated metal centers, creating high-affinity binding sites for PFAS carboxylate and sulfonate groups [29]. For UiO-66, defect engineering via modulator addition (formic acid, acetic acid, or trifluoroacetic acid) has been shown to increase pore accessibility and adsorption capacity for PFOA by up to 200% compared to defect-free analogs [37]. In COFs and MCOFs, controlled introduction of layer vacancies, edge defects, and missing functional groups can create additional adsorption sites and facilitate molecular diffusion through reduced steric hindrance. Advanced characterization techniques—including positron annihilation lifetime spectroscopy (PALS), solid-state NMR, and high-resolution TEM—enable quantitative defect characterization, while computational screening identifies optimal defect densities for specific PFAS targets. The challenge lies in achieving precise control over defect type, concentration, and spatial distribution to maximize performance without compromising structural stability.

Machine learning (ML) and artificial intelligence (AI) are revolutionizing the discovery and optimization of framework materials for PFAS adsorption. High-throughput computational screening of hypothetical MOF, COF, and MCOF structures—using databases containing millions of candidate frameworks—enables the rapid identification of promising adsorbents before laboratory synthesis. ML models trained on existing adsorption data can predict PFAS binding affinities, adsorption capacities, and selectivity based on structural descriptors (pore size, surface area, functional groups, metal identity). Recent studies have demonstrated the application of graph neural networks and random forest algorithms to predict PFOA adsorption energies with mean absolute errors below 0.2 eV [105]. Beyond traditional ML, generative AI models and large language models are emerging as powerful tools for designing novel frameworks tailored to PFAS capture [106]. These models can propose customized functional monomers and predict their performance across diverse environmental conditions, enabling rapid iteration between computational design and experimental validation. The integration of ML with high-throughput robotic synthesis and automated characterization creates a closed-loop materials discovery pipeline that promises to accelerate the development of next-generation PFAS adsorbents.

The disposal of PFAS-laden spent adsorbents poses significant environmental and economic challenges, as incineration or landfilling of contaminated materials risks secondary pollution [97]. The development of dual-functional materials capable of both adsorbing PFAS and degrading them in situ offers an elegant solution to this problem [107]. In this concept, framework materials function as adsorbent–catalyst hybrids: PFAS molecules are first concentrated within the porous structure through adsorption, then subjected to photocatalytic or electrochemical degradation using the framework’s intrinsic or incorporated catalytic functionality [108]. For photocatalytic degradation, porphyrin-based COFs and MCOFs with visible-light-responsive properties have demonstrated the ability to generate reactive oxygen species (ROS) that cleave C–C and C–F bonds, gradually mineralizing adsorbed PFAS [81]. For electrochemical degradation, conductive MOFs and COFs facilitate electron transfer to PFAS molecules, promoting defluorination at applied potentials [109]. Similarly, defect-engineered MOFs incorporating TiO_2_ or ZnO nanoparticles have shown enhanced photodegradation of perfluorinated compounds under UV or visible light. The challenge remains to achieve complete defluorination (i.e., conversion of C–F bonds to inorganic fluoride) while maintaining adsorbent regenerability over multiple cycles [107].

As framework materials approach commercial viability, comprehensive life cycle assessment (LCA) is essential to evaluate their environmental sustainability relative to conventional adsorbents. LCA quantifies environmental impacts across the entire material life cycle—from raw material extraction and synthesis through use-phase performance to end-of-life disposal [110]. For MOFs, COFs, and MCOFs, key impact categories include global warming potential (GWP, kg CO_2_ eq), cumulative energy demand (CED, MJ), and ecotoxicity. Preliminary LCA studies have revealed that the synthesis of MOFs using conventional solvothermal methods can have GWP values 10–50 times higher than those of granular activated carbon (GAC), primarily due to the high energy demand of solvent heating and the use of toxic organic solvents [111]. However, the superior adsorption capacity and regenerability of framework materials can offset these initial burdens through reduced material consumption and extended service life. For COFs and MCOFs, the higher cost and energy intensity of organic monomer synthesis present additional environmental burdens that must be weighed against their superior stability and performance. Emerging green synthesis routes—including room-temperature, water-based, and mechanochemical methods—offer pathways to substantially reduce environmental footprints, with some studies reporting GWP reductions of 60–80% compared to conventional solvothermal synthesis [78]. System-level LCA considering multiple regeneration cycles, end-of-life options (recycling, incineration, or landfilling), and the potential for PFAS recovery or destruction will be essential for informing material selection and guiding sustainable development of framework-based PFAS remediation technologies [110].

## 7. Conclusions

PFAS have emerged as one of the most persistent environmental contaminants, with their extreme stability, documented toxicity, and stringent regulatory limits driving urgent demand for effective water treatment technologies. Conventional adsorbents—activated carbons and ion-exchange resins—exhibit inherent limitations including slow kinetics, poor short-chain removal, and unsatisfactory regeneration. MOFs, COFs, and the emerging MCOFs have demonstrated transformative potential as advanced adsorbents, offering high surface areas, programmable pore architectures, and tunable functionalities that enable tailored host–guest interactions.

Each framework family occupies a complementary niche in PFAS remediation. MOFs offer exceptional synthetic versatility, with atomically precise engineering pushing PFOA adsorption capacities beyond 3000 mg/g, yet their coordination chemistry imposes inherent hydrolytic stability limitations. COFs leverage strong covalent linkages to achieve exceptional chemical stability across pH 2–12, with well-ordered channels enabling rapid kinetics, though the high cost of organic monomers remains a barrier to large-scale implementation. MCOFs represent a paradigm-shifting class that integrates coordinatively bound metal centers within covalent backbones, combining metal-specific binding with hydrolytic stability. Through metal center engineering, the d-band center has emerged as a predictive descriptor for adsorption performance, with Ce-COF demonstrating exceptional PFOA capacity while maintaining robust stability across a broad concentration range.

From a comparative perspective, MOFs excel in applications prioritizing maximum capacity and rapid kinetics in clean water matrices, COFs are the materials of choice for harsh conditions—strongly acidic/basic pH and elevated temperatures—where their exceptional stability ensures sustained performance, while MCOFs, with their balanced profile, offer a versatile intermediate solution and represent the most promising frontier for next-generation adsorbent development. Material selection ultimately requires consideration of the specific application context rather than a universal “best” material.

Looking forward, several critical developments will accelerate technological translation: defect engineering enhances performance by exposing additional open metal sites; machine learning and high-throughput screening accelerate discovery of optimal framework compositions; integration of adsorption with in situ photocatalytic or electrochemical degradation addresses the critical challenge of spent adsorbent disposal; and life cycle assessment is essential to quantify environmental footprints and guide sustainable, scalable synthesis. The convergence of these advances promises to transition MOF, COF, and MCOF adsorbents from academic research to practical water treatment solutions, contributing to the mitigation of PFAS contamination and the protection of global water resources.

## Figures and Tables

**Table 1:** Classification and chemical structures of representative PFAS compounds.

Class ^1^	Compound Name	Abbreviation	Structure	Molecular Weight (g/mol)
Perfluoroalkyl Carboxylic Acid (PFCA)	Perfluorononanoic Acid	PFNA	CF_3_(CF_2_)_7_COOH	464.08
	Perfluorooctanoic Acid	PFOA	CF_3_(CF_2_)_6_COOH	414.07
	Perfluorobutanoic Acid	PFBA	CF_3_(CF_2_)_2_COOH	214.04
Perfluoroalkyl Sulfonic Acid (PFSA)	Perfluorooctane Sulfonic Acid	PFOS	CF_3_(CF_2_)_7_SO_3_H	500.13
	Perfluorohexane Sulfonic Acid	PFHxS	CF_3_(CF_2_)_5_SO_3_H	400.11
	Perfluorobutane Sulfonic Acid	PFBS	CF_3_(CF_2_)_3_SO_3_H	300.10
Perfluoroalkyl Ether Carboxylic Acid (PFECA)	Hexafluoropropylene Oxide Dimer Acid	GenX (HFPO-DA)	CF_3_CF_2_OCF(CF_3_)COOH	330.05
Fluorotelomer Sulfonate (FTS)	6:2 Fluorotelomer Sulfonate	6:2 FTS	CF_3_(CF_2_)_5_(CH_2_)_2_SO_3_H	428.16

^1^ Within each class, compounds are arranged in descending order of perfluorinated carbon chain length.

## Data Availability

No new data were created or analyzed in this study. Data sharing is not applicable to this article.
